# Response of Sunflower (*Helianthus annuus* L.) Leaf Surface Defenses to Exogenous Methyl Jasmonate

**DOI:** 10.1371/journal.pone.0037191

**Published:** 2012-05-18

**Authors:** Heather C. Rowe, Dae-kyun Ro, Loren H. Rieseberg

**Affiliations:** 1 Botany Department, University of British Columbia, Vancouver, British Columbia, Canada; 2 Department of Biological Sciences, University of Calgary, Calgary, Alberta, Canada; 3 Biology Department, Indiana University, Bloomington, Indiana, United States of America; Max Planck Institute for Chemical Ecology, Germany

## Abstract

*Helianthus annuus*, the common sunflower, produces a complex array of secondary compounds that are secreted into glandular trichomes, specialized structures found on leaf surfaces and anther appendages of flowers. The primary components of these trichome secretions are sesquiterpene lactones (STL), a diverse class of compounds produced abundantly by the plant family Compositae and believed to contribute to plant defense against herbivory. We treated wild and cultivated *H. annuus* accessions with exogenous methyl jasmonate, a plant hormone that mediates plant defense against insect herbivores and certain classes of fungal pathogens. The wild sunflower produced a higher density of glandular trichomes on its leaves than the cultivar. Comparison of the profiles of glandular trichome extracts obtained by liquid chromatography–mass spectroscopy (LC-MS) showed that wild and cultivated *H. annuus* were qualitatively similar in surface chemistry, although differing in the relative size and proportion of various compounds detected. Despite observing consistent transcriptional responses to methyl jasmonate treatment, we detected no significant effect on glandular trichome density or LC-MS profile in cultivated or wild sunflower, with wild sunflower exhibiting a declining trend in overall STL production and foliar glandular trichome density of jasmonate-treated plants. These results suggest that glandular trichomes and associated compounds may act as constitutive defenses or require greater levels of stimulus for induction than the observed transcriptional responses to exogenous jasmonate. Reduced defense investment in domesticated lines is consistent with predicted tradeoffs caused by selection for increased yield; future research will focus on the development of genetic resources to explicitly test the ecological roles of glandular trichomes and associated effects on plant growth and fitness.

## Introduction

Plants possess a variety of constitutive and induced defenses against herbivores and pathogens [1]. Constitutive defenses are, in ideal terms, pre-formed defenses that are produced by a plant regardless of external stimuli. Induced defenses are chemical or physical alterations in the plant, following an initial attack or stimulus, that affect subsequent herbivore or pathogen activity. Constitutive defenses are expected to be most important when pest pressure is high and fairly constant, while induced defenses are predicted to play a larger role when pest populations are variable and the costs of resistance are high [2]–[4].

Comparisons of domesticated plants with their wild relatives can be used to evaluate these predictions. Domesticated species have originated rapidly and recently and often exhibit a reduction in physical and chemical defenses [5]. The decline in resistance is thought to result from a growth-defense trade-off, in which constitutive investments in defense are lost in favor of growth and reproduction [6]. However, distinguishing between constitutive and induced defenses can be difficult, as many defense traits are both constitutive and inducible, to an extent that is influenced by both the timing and strength of the induction stimulus. Also, modern improvement programs attempt to re-introduce resistance to specific pests, which may mask growth-defense trade-offs initially associated with domestication.

Sunflower, *Helianthus annuus*, exhibits a typical domestication syndrome [7]. The domesticated sunflower is interfertile with its wild progenitor, also *H. annuus,* and appears to have been domesticated in eastern North America circa 4000 years ago [8], [9]. Comparisons of cultivated and wild sunflower germplasm indicate that the former have increased susceptibility to disease and insect pressures, although genetic background and environmental factors contribute significant variation [6], [10]–[12].

Resistance to insect herbivores in sunflowers is mediated in part by glandular trichomes, which possess a hair-like stalk that terminates in a secretory head that can accumulate secondary compounds. Glandular trichomes occur broadly throughout dicotyledenous plants, but their contents vary among and within taxonomic groups. Defensive plant secondary metabolites (e.g., terpenoids, alkaloids, and phenylpropanoids) are commonly sequestered in specialized structures, such as glandular trichomes, laticifers, and resin ducts, presumably to avoid autotoxic effects. Additional or alternate functions for glandular trichomes in maintaining water balance and protection from UV damage have been proposed [13]–[16]. Various mechanisms of defense mediated by glandular trichome secretions have been demonstrated, including paralysis of insects by alkaloids produced by tobacco, inhibition of insect movement and chewing by acyl sugars produced by some varieties of tomato, and deterrence of insect feeding by menthol derivatives produced by mints [17]–[21]. Specification of trichomes, as understood from studies of trichome development in the model plant *Arabidopsis thaliana*, occurs in the protodermis, and is influenced by phytohormones including jasmonates, cytokinins, and gibberellins [22]. Trichomes are often described as constitutive defenses, as their relatively early developmental origins mean that they cannot be produced rapidly on a mature plant organ in response to an attack. In various plants, glandular trichomes have been shown to be inducible over a longer time scale by external stimuli, including treatment with exognenous methyl jasmonate, mechanical or insect damage, or osmotic stress [14], [23]–[25]. In these cases, increased density of trichomes is observed only on new leaves that emerge after treatment.

Glandular trichomes of Helianthus species primarily contain sesquiterpene lactones (STL) and flavonoids [26]. STL are a structurally-diverse class of secondary compounds derived from the isoprenoid biosynthetic pathway. Production of these compounds is not exclusive to the plant family Compositae, but STL have been reported in the greatest abundance and diversity of structures in this family [27]. STL contain a basic backbone of 15 carbon atoms derived from joining of three 5-carbon isoprene units, but may vary considerably in arrangement of the basic skeleton (linear vs. cyclic, size of the lactone ring) and composition of side chains.

STL are generally bitter-tasting compounds, and many structures show cytotoxicity in experimental settings [28]. Partially because of the relative physiological expense of producing STL, these compounds are presumed to serve valuable anti-herbivore and anti-microbial defense functions in natural environments [29]. Laboratory assays using purified STL from several Helianthus species have demonstrated either feeding deterrence or growth reduction effects of these compounds on specialist and generalist insect herbivores, including sunflower moth (*Homeosoma electellum*), Southern armyworm (*Spodoptera eridania*), and migratory grasshopper (*Melanoplus sanguinipes*) [30], [31]. Within the family Compositae, STL have been proposed as a useful taxonomic tool for elucidating relationships among closely-related taxa [32], [33]. Within the species *Helianthus annuus*, structures of at least a dozen STL have been elucidated. These reports have generally focused on single *H. annuus* varieties [26], [34]–[36], although a comparison of 3 *H. annuus* cultivars reports similar total STL contents [37]. A study of STL from a wild *H. annuus* population in Texas also reports similar structures from a pooled sample [38].

Here we compare glandular trichome density and contents from an *H. annuus* elite oilseed cultivar (HA89) and a wild population (ANN1238). Additionally, both accessions were treated with exogenous methyl jasmonate (MeJA) to induce stress responses related to herbivore attack. We specifically ask whether (1) the cultivated line HA89 exhibits a reduction in the glandular trichome density and STL concentration relative to the wild line, ANN1238; and (2) reductions in these defensive structures and compounds represent a loss of constitutive defenses as predicted by evolutionary theory.

## Methods

### Plant Growth and Treatment

*H. annuus* HA89 is a highly inbred elite oilseed cultivar developed in Texas and released by the USDA and the Texas Agricultural Experiment Station in 1971. ANN1238 was collected at the University of Nebraska's Cedar Point Biological Research Station and donated to the USDA germplasm collection in 2009. Twenty seeds each of *H. annuus* ANN1238 and HA89 (USDA accessions: PI 659440, PI 599773) were scarified and incubated in darkness at room temperature on moist filter paper. Seeds were checked daily for germination, determined as protrusion of the radicle by at least 2 mm. Seed coats were manually removed from imbibed or germinated seeds to reduce fungal infection. At 3 days post-scarification, 12 seedlings per group were transferred to Promix B soil in 32-cell flats (approximately 100 cm^3^ soil volume/cell) and placed in a controlled environment chamber at 26°C, 50% RH, and 14 h day length. Seedlings were sub-irrigated every other day with tap water.

At two weeks post-germination, seedlings were transplanted to 1-gallon (3.78L) pots containing a 7.4∶4: 1 mixture of peat: perlite: calcined clay, with addition of dolomite 65AG (0.12% volume), Micromax micronutrient mix (0.06% volume), AquaGro 2000 wetting agent (0.01% volume), and Nutricote18-6-8 fertilizer (0.3% volume). Plants were grown in a greenhouse with supplemental lighting for 12 h per day. Plants in different treatment groups were spatially separated on a single greenhouse bench (approximately 5 m separation, with non-treated buffer plants occupying the intervening space). Plants were rotated between bench ends twice-weekly to diminish positional effects due to spatial variation in temperature or lighting.

One of the second pair of fully-expanded true leaves was removed from each plant prior to treatment to evaluate baseline variance within and between groups. This leaf was then photographed, sampled for later RNA extraction, and the density of glandular trichomes estimated (see below). One of the 5^th^ pair of true leaves was sampled from all plants following their third experimental treatment and analyzed similarly to evaluate differences in glandular trichome density, chemical profile, and mRNA transcript accumulation attributable to methyl jasmonate treatment.

**Figure 1:**
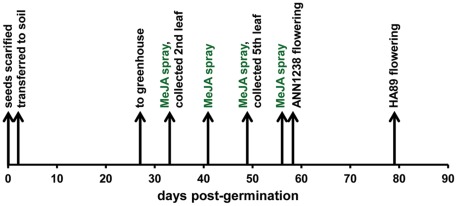
Timeline of *H. annuus* plant growth and treatment.

Whole plants were treated with a foliar spray of 100 µM methyl jasmonate (MeJA) in 0.01% EtOH or a control solution of 0.01% EtOH. MeJA solution was prepared by mixing 100 µmoles of methyl jasmonate (Sigma-Aldrich Inc., Ontario CAN) with 100 µl EtOH to improve solubility in H_2_O, followed by dilution in 1 L filtered H_2_O. Plants were removed from the greenhouse for treatment to avoid contamination of control plants by volatile MeJA, and returned approximately 1 hour after spraying. A timeline of plant treatments and sampling is provided as Figure 1.

In a separate experiment, seedlings of both accessions were germinated and transplanted to soil as described above. One week following transplant to soil, seedlings were treated with 1 mM MeJA in 0.01% EtOH or control treatment (0.01% EtOH), also as previously described. Plants were placed in clear plastic bags for treatment and two hours following, then the bags were removed. The plants were allowed to dry for an additional hour before returning to the greenhouse bench. This treatment was repeated the following week, prior to transplanting 2 week-old plants into 1-gallon pots. At 4 weeks post-germination, one of the second pair of true leaves was removed from each plant for estimation of glandular trichome density and leaf area. At the same time, plant height (from base of soil, in cm) was measured.

### Foliar Glandular Trichome Density

Density of glandular trichomes on the abaxial leaf surface was determined by counting the number of glandular trichomes contained in a 3 mm×3 mm ocular grid, observed at 25×magnification, for three regions of the leaf, roughly delineated as leaf tip, leaf edge, and leaf center proximal to midvein. Although we attempted to harvest only fully-expanded leaves, the average number of glandular trichomes per cm^2^ was multiplied by the leaf area to diminish the effect of any variation in leaf expansion. The fate of cells destined to become glandular trichomes is determined early in development, thus the number of trichomes per leaf should remain constant even as the leaf area increases through expansion. Analyses of variance using generalized linear models in R tested the explanatory value of parameters ‘ACCESSION’ (“HA89”, “ANN1238”), TREATMENT (“control”, “MeJA”), and their interaction to explain observed variation in trichome counts.

### Extraction of Glandular Trichome Contents

Following determination of glandular trichome density, leaves were rinsed in HPLC-grade dichloromethane (Fisher Scientific) for 15 seconds. This extract was dried under air, resolved in 100% MeOH, and stored at −80°C until analysis. Flowers were collected from each plant when the first flower was fully open (ray flowers fully-expanded) and the anther appendages of the outside ring of disk flowers were extended past the corolla (Figure 1). Approximately 150 glandular trichomes were removed from the anther appendages with a flat-tipped needle and collected into a 1.5 ml centrifuge tube containing 0.5 ml HPLC-grade methanol. These samples were dried in a vacuum-centrifuge and stored at −80°C until analysis.

### Analysis of Glandular Trichome Profiles by Liquid Chromatography-Mass Spectroscopy (LC-MS)

LC-MS analysis was performed using an Agilent 1200 Rapid Resolution LC (RRLC) system coupled with Agilent 6410 MS. Two µL of samples were separated through a Zorbax Eclipse plus C18 (2.1×50 mm, 1.8 µm) column at 40°C. Solvents used were water (with 1% acetic acid) and acetonitrile (100%). The acetonitrile composition was increased from 10% to 80% over 20 min with a 30 sec holding time at the start (a total of 20.5 min run time). Electrospray ionization method was used, and signals for both positive and negative ions were collected to infer the masses of eluted STL. Qualitative analysis was performed using Agilent MassHunter Workstation Software (Version B.02.00).). Using a chromatogram showing total mass counts, peaks with retention times between 4–12 minutes were manually integrated and mass spectra were extracted for these peaks. Peaks sharing similar retention time and mass spectra were grouped for comparative analysis of peak area via linear modeling in R (Table 1). Both raw peak areas and values standardized by the estimated number of trichomes sampled ( =  estimated glandular trichomes/leaf; mean of glandular trichome density (trichomes/cm^2^) x leaf surface area (cm^2^) were compared. Linear models tested the explanatory value of parameters ‘ACCESSION’ (“HA89”, “ANN1238”), TREATMENT (“control”, “MeJA”), and their interaction to explain observed variation in peak area.

**Table 1:** Peaks detected in extracts of *H. annuus* glandular trichomes.

					PERCENT OF SAMPLE^c^				
LABEL	RT^a^	mass^b^	compounds^b^		HA89	ANN1238			
A	4.1	266	unknown		0.6–1.2	1.6–2.7			
B	6.0	394	Niveusin A		8.1–12.6	6.5–10.6			
C	6.4	396	4,5-dihydroniveusin A		4.9–11.3	6.1–10.8			
D	6.7	380	Argophyllin B		3.2–9.3	10.6–19.6			
E	7.0	378	1,2-anhydro-4,5-dihydro-niveusin A		1.9–5.6	1.6–3.4			
F	7.3	380	unknown STL		2.0–3.6	0.3–1.1			
G	7.5	376	15-hydroxy-3-dehydrodesoxytifruticin		22.4–30.6	20.6–29.1			
H	8.1	410	1-methoxy-4,5-dihydroniveusin A	4.7–11.3			6.3–12.2		
I	8.7	378	Niveusin B or C	4.8–12.7			2.3–4.4		
JŦ	9.1	408/410	Methoxy derivatives of two STL	12.1–16.1			15.3–18.3		
K	9.6	360	unknown	1.7–3.4			2.3–7.2		

a Retention time (minutes)

b masses of compounds inferred from (+/−)-LC-MS data. The mass to charge ratio (*m/z*) for [M−H]^-^, [M + Cl]^-^, or [M + acetic acid]^-^ ions were detected in (−)-LC-MS, whereas the *m/z* for [M+H]^+^, [M+Na]^+^, or [M+K]^+^ ions were detected in (+)-LC-MS. When the compounds were named, the masses and the eluting patterns in a C18 column (i.e., hydrophobicity of STLs) were considered. The separation pattern detected in a (−)-LC-MS was given as a reference in Figure 3. Published papers [35], [36] were used to guide these chemical identifications.

c range of values (n = 6 samples/accession) for the percent contribution of this sample peak to total peak area measured per sample.

Ŧ Indicates peaks containing two high-abundance *m/z* values, suggesting multiple co-eluting compounds.

### Transcriptional Response to Jasmonate Treatment

Tissue for RNA extraction was sampled from leaves from leaf pairs 2 (pre-treatment) and 5 (post-treatment). Leaf 2 was sampled immediately following excision from the plant and prior to assessment of glandular trichome density. Leaf 5 was sampled 24 hours after treatment with MeJA; plants had received three MeJA treatments at this point (Figure 1). Eight leaf discs of approximately 6 mm in diameter (∼50 mg fresh weight) were removed from each leaf immediately following excision of the leaf from the plant and flash-frozen in a 1.5 ml tube in liquid nitrogen. Tissue was stored at −80°C until extraction of total RNA using Trizol (Invitrogen) as described [39].

Further sample preparation and whole transcriptome shotgun sequencing of cDNA derived from these samples was performed at the Michael Smith Genome Sciences Centre in Vancouver, British Columbia, Canada (http://www.bcgsc.ca/services). Unaligned sequence files in BAM format were converted to fastq format using bam2fastq (www.hudsonalpha.org/gsl/software/bam2fastq.php). Reads passing the quality control threshold imposed by Illumina pipeline defaults (chastity >0.6) were aligned to a transcriptome reference compiled from 93428 EST sequences from several *H. annuus* accessions [40], [41], containing 16312 unique contigs. Alignments were performed using the Burrows-Wheeler Aligner (BWA) tools ‘aln’ and ‘sampe’ [42]. Aligned BAM files were sorted and PCR duplicates removed using SAMtools utilities ‘sort’ and ‘rmdup’ [43]. Reads per contig were counted for each sample using coverageBed [44]. Read counts were analyzed in R using the DESeq package to compare within-transcript read counts [45]. Specific pairwise comparisons between timepoints (pre-treatment and post-treatment) for treated samples and between treatments (control and MeJA) for post-treatment samples were conducted within each accession. Contigs showing significant differences (FDR-adjusted p-value <0.01) in transcript accumulation in multiple independent comparisons were used as queries to search the NCBI non-redundant nucleotide database for similar sequences via the BLASTN (version 2.2.26+) discontinuous megablast algorithm [46].

## Results

### ANN1238 Produced a Higher Density of Foliar Glandular Trichomes than HA89 Across All Leaf Positions, Time Points, and Treatments Assessed (Figure 2)

**Figure 2:**
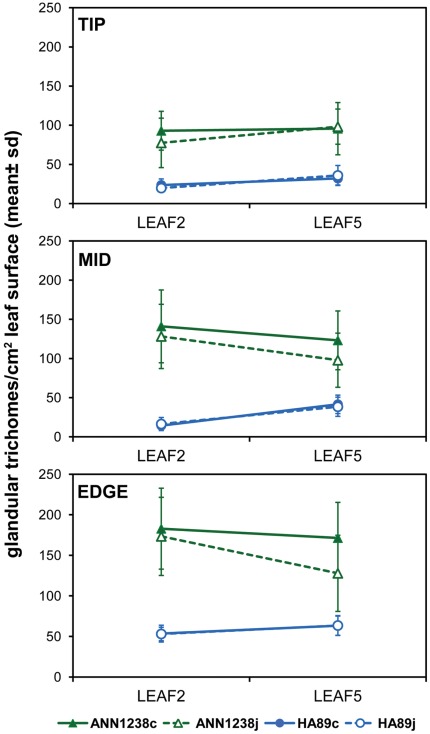
Glandular trichome count per 3 mm×3 mm grid of *H. annuus* abaxial leaf surface. ‘LEAF2’ was removed from the plant prior to the first methyl jasmonate treatment; ‘LEAF5’ was removed two weeks later, after two treatments with 0.1 mM methyl jasmonate (MeJA) or control spray spaced at one-week intervals. Accession is indicated by marker shape (▴ =  ANN1238, •  =  HA89) and treatment is indicated by filled (control) versus open (MeJA) markers. Data points represent the mean value from 6 plants; error bars show one standard deviation. Where data points from both treatments are not clearly visible (e.g. HA89 EDGE), they are overlapping.

Glandular trichome density was estimated at 3 positions on the leaf: leaf tip, mid-leaf, and lower edge. Both accessions produced the highest estimated density of glandular trichomes at the lower leaf edge. For ANN1238, however, mid-leaf glandular trichome density estimates were similar to those from the lower leaf edge, while estimates from the mid-leaf of HA89 were lower than glandular trichome density estimates from the leaf tip. Within accession, mean foliar glandular trichome density did not significantly differ between the second and fifth leaves. HA89 leaves from both collection times had significantly larger surface area and were more circular in shape than ANN1238 leaves.

### No Significant Effect of Exogenous MeJA Treatment on Density of Foliar Glandular Trichomes on the Fifth Leaf was Observed (Figure 2)

Plants assigned to the MeJA treatment were sprayed with 100 µM MeJA following removal of one of the second leaf pair for estimation of initial glandular trichome density (Figure 1). After 2 weeks and two additional applications of MeJA, one of the 5^th^ pair of true leaves was removed for estimation of glandular trichome density. Analysis of variance revealed no significant difference in glandular trichome counts between treated and untreated plants at any position (Figure 2). A marginally significant effect of the interaction between *H. annuus* accession and treatment is explained by lower glandular trichome counts on the leaf edge observed in jasmonate-treated ANN1238 (p = 0.09). Leaves from jasmonate-treated plants from both accessions had similar surface area to controls.

Plants of both accessions showed strong inhibition of growth in response to a 1 mM MeJA treatment applied for two weeks to one and two week-old plants (Figure S1). The second true leaf of plants subjected to this high dosage of MeJA had significantly higher density of glandular trichomes, but this was entirely explained by the dramatically reduced surface area of the leaf in comparison to the second true leaf from control group plants. When leaf surface area was multiplied by trichome density to estimate glandular trichomes per leaf, MeJA-treated and control group leaves of both accessions displayed no significant difference in glandular trichome content, as observed for lower dosage MeJA treatments described above.

### Little Variation in STL Profiles was Detected within Accessions

**Figure 3:**
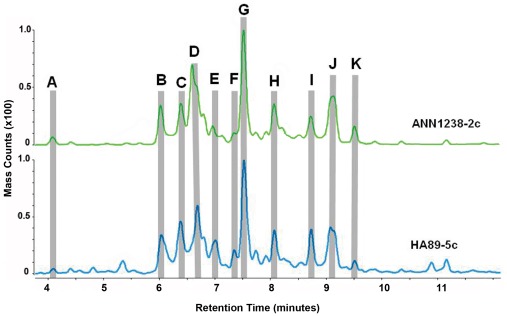
Representative chromatograms of methylene chloride extracts from the leaf surfaces of *H. annuus* accessions ANN1238 and HA89. The horizontal axis displays retention time in minutes. Vertical axes are scaled identically and display total mass counts. Labeled peaks A-K were selected for further analysis and comparison between accessions, and correspond to similarly-labeled peaks in Table 1 and Figures 4 and 5.

Eleven peaks were identified in all leaf wash samples analyzed (Table 1, Figure 3). These peaks accounted for 88–97% of analyzed peak area per sample. Wild accession ANN1238, although expected to possess higher genetic and phenotypic variability than the highly inbred HA89, showed no qualitative variation in peaks detected and proportional contributions within glandular trichome profiles among the 10 individuals analyzed. ANN1238 samples did, as a group, have higher standard deviation in areas of several peaks, notably peaks G and J, however this pattern may be attributable to higher variation in glandular trichome density, as HA89 actually showed higher variance in standardized peak areas.

**Figure 4:**
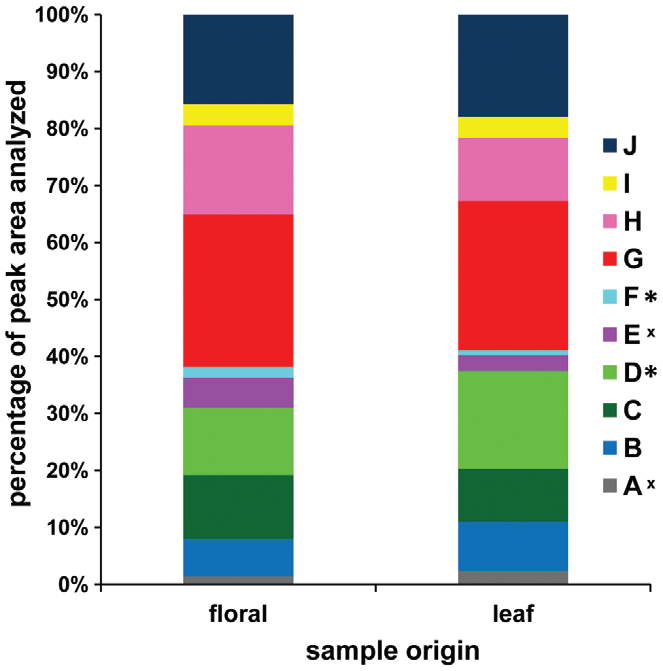
Relative percentages of LC-MS peak area accounted for by 10 peaks shared between glandular trichome extract profiles derived from floral (anther appendage) versus leaf (abaxial surface) samples. Relative peak percentage is determined as (peak area)/(sum of areas for peaks A-J). Statistical significance of differences among sample sources are marked in the legend: * = p<0.05, ^x^ = p<0.1. Peaks are marked in Figure 3; retention time and neutral mass for each peak are given in Table 1.

**Figure 5:**
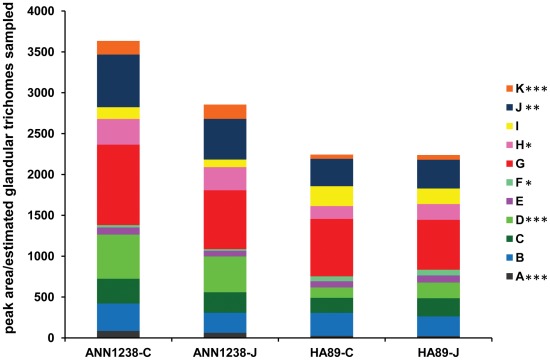
Comparison of common peaks from *H. annuus* foliar glandular trichome LC-MS profiles. Samples are grouped by accession (ANN1238, HA89) and treatment (C  =  control, J  = 100 µM MeJA) along the horizontal axis. The vertical axis shows the peak area (mass counts) standardized by the estimated number of glandular trichomes extracted per sample (cm^2^ leaf area *average glandular trichomes/cm^2^). Each colored section within the columns corresponds to a specific peak, indicated in the legend and corresponding to Table 1. Values shown represent the mean peak area for 5 samples per accession x treatment; statistically significant differences between HA89 and ANN1238-derived samples are marked in the legend: * = p<0.05, ** = p<0.01, *** = p<0.001).

### Flower-derived Glandular Trichome Profiles were Similar to those Derived from Leaf Washes (Figure 4)

Glandular trichomes were manually removed from anther appendages of HA89 and ANN1238 florets at the stage where the anther tube had extended from the corolla but the anthers had not yet emerged. These samples were extracted directly with 100% MeOH, in contrast to leaf samples, which were washed with CH_2_Cl_2_, dried, and re-suspended in MeOH. Despite this difference in extraction methods, as well as a substantial difference in the volume of material extracted (∼150 glandular trichomes/floral sample vs. 1–5×10^4^ estimated glandular trichomes/leaf abaxial surface), LC-MS profiles of glandular trichome samples derived from leaves and flowers were qualitatively similar. All peaks listed in Table 1 were observed in LC-MS profiles of floral glandular trichome extracts, with the exception of Peak K. Comparison of the relative proportion of glandular trichome extract profile accounted for by individual peaks showed that the relative proportions of peaks D and F differed between floral and leaf samples, with D accounting for a relatively smaller portion of floral extracts (Figure 4).

### Likely STL Identities

The chemical identities of the STL from ANN1238 and HA89 were inferred by comparing the mass data from LC-MS to the previously published structural data [35], [36] (Table 1). The major STL identified were niveusin A (peak B), 4,5-dihydroniveusin A (peak C), argophyllin B (peak D), 15-hydroxy-3-dehydrodesoxytifruticin (peak G), and Niveusin B/C (peak I). Differences in retention time among peaks sharing the same mass is likely to reflect structural rearrangement. Peaks A and K are unknown compounds that do not match the mass or elution patterns of any described STL. As peak K is not observed in the floral-derived glandular trichome profiles, it seems likely that this peak represents a compound washed from the leaf surface, rather than glandular trichome contents.

### Significant Differences were Detected in the Relative Peak Areas of Compounds Produced by Both ANN1238 and HA89 (Table 1)

While absolute quantification is not feasible without reference standards, the mass counts of matching peaks can becompared among samples. Area of each peak analyzed was standardized by the estimated number of glandular trichomes sampled; for leaf washes, this was approximated as the number of glandular trichomes on the total washed leaf surface (average glandular trichomes/cm^2^ * cm^2^ leaf area). Total peak area of the 11 compounds chosen for analysis was significantly greater for ANN1238 samples than HA89. Within the analyzed profile, peaks A, D, H, J, K were represented by significantly greater peak areas in ANN1238 samples, with only peak F significantly larger in HA89 samples. Because the observed density of glandular trichomes is greater on ANN1238 leaves than HA89 leaves, the difference in actual production of STL by these accessions is likely greater than implied by Figure 5. Within accessions, averaged profiles of HA89 were nearly identical between control and MeJA-treated plants. ANN1238 samples showed a non-significant decrease in peak areas observed in extracts from MeJA-treated plants.

### Both *H. annuus* Accessions showed Transcript Accumulation Responses to MeJA Treatment

**Figure 6:**
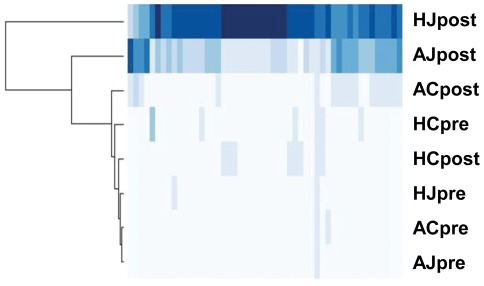
Heatmap showing normalized transcript accumulation of putative jasmonate-responsive transcripts in *H. annuus* samples: ‘H’  =  HA89, ‘A’  =  ANN1238, ‘J’  =  MeJA treatment group, ‘C’  =  control group, ‘pre’  =  pre-treatment, ‘post’  =  post-treatment. Fifty *H. annuus* transcripts showing significant positive response to exogenous MeJA across multiple comparisons are arrayed in columns with color intensity indicating transcript abundance. A list of transcripts and associated annotation by best blast hit is provided in Table S1. The dendrogram on the left indicates clustering by similarity of transcript patterns among samples.

Consistent differences in transcript abundance were observed between jasmonate-treated and control plants (Figure 6). Sets of transcripts were identified as significantly different after adjustment for multiple testing in six pairwise comparisons (within-accession HA89 or ANN1238: control pre-treatment vs. jasmonate pre-treatment, jasmonate pre-treatment vs. jasmonate post-treatment, and control post-treatment vs. jasmonate post-treatment). Transcripts identified as significantly differing between control pre-treatment and jasmonate pre-treatment samples were considered to represent an empirical estimate of false positive results due to uncontrolled biological differences among plants assigned to differing treatment groups, and were removed from the final transcript set. Comparison of the transcript lists generated by the remaining four comparisons revealed 66 transcripts consistently significantly differing in accumulated mapped sequence reads (Table S1). Of these, 48 transcripts were significantly more abundant in jasmonate-treated samples (Figure 6). FastA-formatted reference sequence for these transcripts is provided as Supplemental Data (Data S1). This set included several transcripts with highly-significant sequence similarity to transcribed genes whose functional annotations are directly or indirectly related to jasmonate or defense responses, including 2 lipoxygenases, an ethylene response factor, 2 pathogenesis-related proteins and 3 WRKY transcription factors [47]. In addition, samples from MeJA-treated plants showed significantly greater accumulation of several transcripts similar to published gene sequences from terpene and flavonol biosynthetic pathways (Table S1).

## Discussion

The partitioning of finite resources in biological systems should result in trade-offs between allocation to different aspects of organismal life history, such as growth and defense. Strong selection for increased yield imposed during plant domestication is predicted to result in reduced plant allocation to defense, either through trade-offs in resource allocation or reduced selection to maintain defenses in relatively benign agricultural environments [5], [48]. This prediction has received empirical support from several studies, including comparison of insect performance on teosintes versus maize and insect performance and glucosinolate production in wild, feral, and cultivated Brassica species, in which domesticated plants show significantly lower levels of defense [49], [50].

Within *H. annuus*, domesticated accessions have been shown to be more susceptible to the fungal pathogen *Botrytis cinerea* and more palatable to insect herbivores than wild accessions [6]. Weedy *H. annuus* occupying agricultural habitats have shown transcriptional downregulation of genes associated with defense or biotic stress tolerance when compared to wild populations [51]. The specific accessions used in this study, HA89 and ANN1238, have been used to generate structured mapping populations employed in field studies of herbivory. For 6 of 7 QTL associated with leaf or head herbivory detected in field experiments using a population of recombinant inbred lines generated from HA89 x ANN1238, the ANN1238 allele conferred reduced levels of herbivory [52].

In this study, we find that cultivated *H. annuus* HA89 possesses a lower density of foliar glandular trichomes than wild accession ANN1238 (Figure 2). Comparison of glandular trichome extracts from these accessions indicates that similar structures are produced in both accessions, yet qualitative differences in the relative proportions of some compounds produced were observed (Figure 5). In particular, higher production of Argophyllin B in glandular trichome extracts of ANN1238 versus HA89 may indicate polymorphism between these accessions in a regulatory factor influencing STL production. HA89 samples possessed lower per-trichome estimates of peak areas for 5 of 6 compounds found to significantly differ in amount produced between HA89 and ANN1238, supporting an overall reduction in investment in STL as a constitutive defense in this accession.

### Surprising Consistency of Glandular Trichome Profiles

STL exhibit high levels of structural variation across species or genera, to an extent that they have been proposed as useful taxonomic characters. To date, no formal intraspecific comparisons of STL profiles have been published for sunflowers. In this study, little variation in chemical profiles of glandular trichome contents was observed within or between accessions. For HA89, a highly inbred elite cultivar, it may be proposed that the uniformity of chemical profiles is explained by the lack of genetic variation within this accession. ANN1238, however, contains high levels of polymorphism and heterozygosity typical of wild *H. annuus* populations [53], [54]. Yet we observed little variation in chemical profiles of glandular trichome contents among individuals of this accession. While current data do not allow us to determine levels of quantitative variation in the overall levels of STL and flavonoids produced, all individuals of ANN1238 assessed showed strong qualitative similarity in the profile peaks produced and relative peak proportions within the LC-MS profiles of glandular trichome extracts.

Intraspecific structural variation in chemical defenses has been thoroughly documented in Arabidopsis species, where structural variation in glucosinolate production has been empirically linked to fluctuating herbivore community composition [55]–[57]. The consistency of glandular trichome STL profiles within and between the *H. annuus* accessions observed in this study is surprising since chemical defences have frequently been shown to be under balancing selection in other systems such as Arabidopsis [58].

### Glandular Trichomes: A Constitutive Defense?

Glandular trichomes are generally proposed to function as defenses against or deterrents to herbivory. The contents of sunflower glandular trichomes are produced via specialized metabolic pathways (i.e., cannot rationally be considered as “waste products”) and have demonstrated cytotoxicity [28], [59]. Various STL extracted from wild Helianthus species have been demonstrated to inhibit insect feeding or growth, including the compound Argophyllin B, produced by H. annuus as well as H. argophyllus [30], [31]. *H. annuus* STL are also reported to have inhibitory effects on western corn rootworm feeding, and neurotoxic effects when injected into caterpillars [60].

In a spectrum of plant defense responses, trichomes are generally considered to be a constitutive structural defense. Due to their early specification during leaf development, glandular trichomes cannot be rapidly induced. Observation of trichome induction in various plants requires formation of new leaves following the initial stimulus [61], [62]. Treatment effects on glandular trichome density may also be confounded with phenological changes. While phenology of glandular trichome density has not been explored in *H. annuus*, here we observed no significant change in glandular trichome density between the 2^nd^ and 5^th^ leaves of either accession (Figure 2). It may be predicted that leaves produced closer to flowering might show greater investment in chemical defense. Similar reasoning would predict greater investment in chemical defenses on floral structures, only qualitative comparison of floral and leaf investment in glandular trichomes and their contents was feasible in this study (Figure 4).

Treatment of plants with synthetic hormones can provide valuable information about plant signaling pathways and regulation of responses to the environment, particularly in species lacking isogenic lines with identified mutations in hormone-mediated signaling or hormone biosynthetic pathways. Responses to these treatments often vary among or within plant species, and phenotypes may not respond linearly to increased hormone dosage. We observed consistent transcriptional differences between MeJA-treated and control plants, both within the same plant (comparing pre-treatment and post-treatment samples) and between treated and control plants sampled at the same time (Figure 6, Table S1). Strong sequence similarity of these consistently MeJA-responsive transcripts to described jasmonate-response and defense-related genes affirms that both *H. annuus* accessions respond to exogenous methyl jasmonate in a manner at least partially predictable from studies of model plant systems such as *Arabidopsis thaliana*.

No increase in the density of foliar glandular trichomes or significant alteration in their chemical profile was observed on the 5^th^ true leaf of *H. annuus* treated with 0.1 mM methyl jasmonate (Figures 2 and 5). In fact, wild *H. annuus* accession ANN1238 showed a non-significant trend toward decreased density of foliar glandular trichomes and relative decreases in STL content in MeJA-treated plants versus controls. It is possible that the dosage of MeJA was insufficient to induce increases in glandular trichome density or alterations in chemical profiles. This would imply that *H. annuus* is less sensitive to exogenous MeJA treatment than *Arabidopsis thaliana* or *Artemisia annua* [63], [64]. Increasing the dosage of MeJA to 1 mM led to strong reduction in plant growth for both accessions observed (Figure S1). Observed increases in trichome density in these plants corresponded with 61 and 74 percent reduction in mean leaf surface area for HA89 and ANN1238 respectively; when leaf area is taken into account the total estimated production of foliar glandular trichomes does not significantly differ among treatments, as observed with the 100 uM MeJA treatments. It would be interesting to test whether increased density of glandular trichomes achieved via reduction in leaf size may affect the ability of insect herbivores to avoid contact with plant defenses.

*H. annuus* glandular trichomes and associated STL might act primarily as constitutive defenses, with other, more rapidly mobilized defenses that respond to MeJA. This hypothesis is consistent with findings from Eucalyptus species, where MeJA treatment had no observable effects on foliar terpene content [65]. Intriguingly, several transcripts with strong sequence similarity to terpene synthases were significantly more abundant in MeJA treated plants (Table S1). These transcripts do not encode terpene synthases linked to STL biosynthesis in sunflower trichomes and are not listed in the sunflower trichome-specific EST database (767 unigenes) [66], [67]. As the specific biosynthetic functions of these genes are currently unknown, it is likely that these transcripts contribute to production of MeJA-induced terpenes that are not secreted into glandular trichomes or are rapidly volatilized from the leaf surface. Thus, in sunflower, STL in trichomes may serve as constitutive defenses while other types of terpenes are responsive to external stimuli as part of an induced defense mechanism. Further experiments using increased concentrations of MeJA or greater frequency of treatments may determine whether higher dosage or more frequent MeJA treatments can significantly alter STL profiles, although growth repression or toxic effects in the plant caused by high dosages of MeJA may reduce the value of such studies for predicting ecologically-relevant effects [68], [69] (Figure S1).

Several studies observing glandular trichome induction over a period of plant growth reported a saturation of induction, i.e. a time point where the maximum glandular trichome density is observed, with subsequently produced leaves showing similar or lower glandular trichome densities [23], [24]. Therefore, an additional explanation of the results observed in this study is that both *H. annuus* accessions had reached a threshold of glandular trichome production induced by common environmental factors.

### Future

The accessions examined in this study, HA89 and ANN1238, are also the parents of an established population of recombinant inbred lines that has served as a valuable resource for identification of regions of the *H. annuus* genome correlated with agriculturally-important phenotypes. Observations of large, genetically controlled differences in production of glandular trichomes between these accessions suggest the future possibilities of using this population to identify genomic regions responsible for these differences and develop nearly isogenic lines as a resource to test the effects of glandular trichome density on plant stress responses.

## Supporting Information

Figure S1 **Response of** ***H. annuus*** **accessions HA89 and ANN1238 to 1 mM MeJA.** A) Growth inhibition by MeJA treatment is indicated by the reduced stature of MeJA-treated plants compared to control. Plants shown are 4 weeks old, following treatment with 1 mM MeJA or control solution at 1 and 2 weeks post-germination. Treatments and accessions are as labeled in the photo. B) Significant differences in plant height, leaf area, and density of glandular trichomes, but not in estimates of total number production of foliar glandular trichomes were observed in response to MeJA treatment. Values provided are ‘mean (standard deviation)’ for 6 plants per (treatment x accession). The last 3 columns indicate statistical significance of the terms ‘ACCESSION’ (ANN1238 vs. HA89), ‘TREATMENT’ (control vs. MeJA), and ‘INTERACTION’ (TREATMENT × ACCESSION) within an analysis of variance model: ‘*’p<0.05, ‘***’p<0.001.
(PDF)

Table S1 **List of transcripts showing consistent statistically-significant differences in accumulation among four sample comparisons: ANN1238 post treatment MeJA vs. CTRL, HA89 post treatment MeJA vs. CTRL, ANN1238 pre-treatment vs. post-treatment (MeJA), HA89 pre-treatment vs. post-treatment (MeJA).** “reference contig” identifies the reference sequence provided as Data S1 (Reference Transcripts). “set” identifies transcript groups as “JA induced” (higher accumulation in MeJA-treated plant samples), “JA repressed” (lower accumulation in MeJA-treated plant samples), or “bias” (showing statistically significant differences in accumulation between plants assigned to CTRL vs. MeJA groups prior to experimental treatment). “AJ-AC(post)”, “HJ-HC(post)”, “AJ(po-pre)”, “HJ(po-pre)” provide the mean difference in transcript accumulation for each comparison. “best BLAST hit” provides the GenBank identifier for the most similar sequence in the NCBI nucleotide database as of January 2012. “blasthit_summary” sumarizes the available annotation for the top 10 BLAST hits for this transcript. “evalue” estimates the significance of the top BLAST hit; this table is also color-coded to indicate transcript levels in MeJA samples (blue  =  lower, green  =  higher), with depth of color indicating confidence in similarity to annotated sequence (lighter  =  higher evalue and lower BLAST score).
(PDF)

Data S1 **FastA-formatted reference sequence for** ***H. annuus*** **methyl jasmonate responsive transcripts listed in Table S1.**
(TXT)
