# Supplementary material for: Response of Sunflower (Helianthus annuus L.) Leaf Surface Defenses to Exogenous Methyl Jasmonate
Source: PLoS One. 2012 May 18;7(5):e37191. doi: 10.1371/journal.pone.0037191 (PMC3356381; doi:10.1371/journal.pone.0037191)
Supplement: Figure S1 — Response of H. annuus accessions HA89 and ANN1238 to 1 mM MeJA. A) Growth inhibition by MeJA treatment is indicated by the reduced stature of MeJA-treated plants compared to control. Plants shown are 4 weeks old, following treatment with 1 mM MeJA or control solution at 1 and 2 weeks post-germination. Treatments and accessions are as labeled in the photo. B) Significant differences in plant height, leaf area, and density of glandular trichomes, but not in estimates of total number production of foliar glandular trichomes were observed in response to MeJA treatment. Values provided are ‘mean (standard deviation)’ for 6 plants per (treatment x accession). The last 3 columns indicate statistical significance of the terms ‘ACCESSION’ (ANN1238 vs. HA89), ‘TREATMENT’ (control vs. MeJA), and ‘INTERACTION’ (TREATMENT × ACCESSION) within an analysis of variance model: ‘*’p<0.05, ‘***’p<0.001. (PDF) [file pone.0037191.s001.pdf]

A

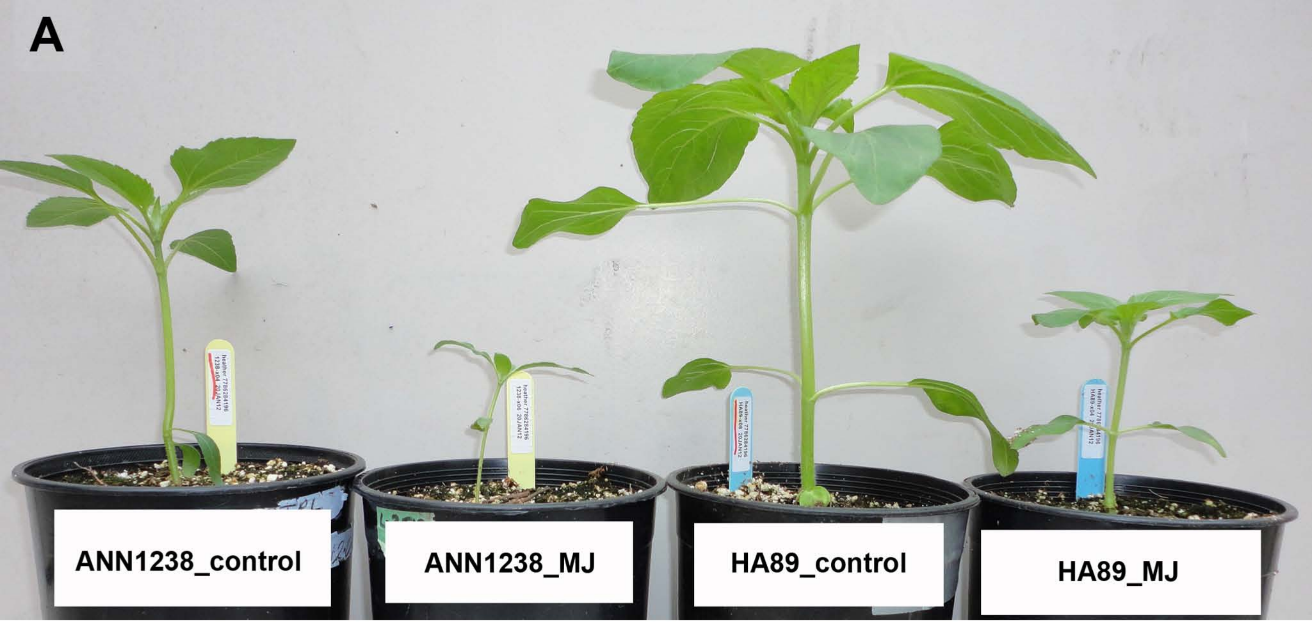

B

TRAIT	ANN1238_control	ANN1238_MJ	HA89_control	HA89_MJ	ACCESSION	TREATMENT	INTERACTION
HEIGHT (cm)	13.72 (4.02)	6.98 (0.89)	15.48 (2.67)	13.5 (4.66)	*	*	NS
LEAF AREA (cm^2)	11.56 (4.37)	2.96 (0.66)	37.82 (6.96)	14.62 (3.78)	***	***	**
GLANDULAR TRICHOMES (density, per cm^2)	716.3 (114.7)	2102.2 (432.3)	224.4 (57.3)	544.4 (81.5)	***	***	***
GLANDULAR TRICHOMES (estimated per leaf)	8213.1 (3083.7)	6004.9 (597.2)	8462.4 (2375.3)	8136.4 (2891.5	NS	NS	NS
