# Supplementary material for: Response of Sunflower (Helianthus annuus L.) Leaf Surface Defenses to Exogenous Methyl Jasmonate
Source: PLoS One. 2012 May 18;7(5):e37191. doi: 10.1371/journal.pone.0037191 (PMC3356381; doi:10.1371/journal.pone.0037191)
Supplement: Table S1 — List of transcripts showing consistent statistically-significant differences in accumulation among four sample comparisons: ANN1238 post treatment MeJA vs. CTRL, HA89 post treatment MeJA vs. CTRL, ANN1238 pre-treatment vs. post-treatment (MeJA), HA89 pre-treatment vs. post-treatment (MeJA). “reference contig” identifies the reference sequence provided as Data S1 (Reference Transcripts). “set” identifies transcript groups as “JA induced” (higher accumulation in MeJA-treated plant samples), “JA repressed” (lower accumulation in MeJA-treated plant samples), or “bias” (showing statistically significant differences in accumulation between plants assigned to CTRL vs. MeJA groups prior to experimental treatment). “AJ-AC(post)”, “HJ-HC(post)”, “AJ(po-pre)”, “HJ(po-pre)” provide the mean difference in transcript accumulation for each comparison. “best BLAST hit” provides the GenBank identifier for the most similar sequence in the NCBI nucleotide database as of January 2012. “blasthit_summary” sumarizes the available annotation for the top 10 BLAST hits for this transcript. “evalue” estimates the significance of the top BLAST hit; this table is also color-coded to indicate transcript levels in MeJA samples (blue = lower, green = higher), with depth of color indicating confidence in similarity to annotated sequence (lighter = higher evalue and lower BLAST score). (PDF) [file pone.0037191.s002.pdf]

reference contig	set	AJ-AC(post)	HJ-HC(post)	AJ(po-pre)	HJ(po-pre)	best BLAST hit	blasthit_summary
BigSet013477|Contig45223	JA induced	51.3	1242.3	56.3	1292.7	gi|6469615|gb|AF154124.1|AF154124	terpene biosynthesis
BigSet009241|Contig13600	JA induced	105.0	594.0	109.0	653.3	gi|4099832|gb|U90265.1|ZEU90265	nuclease
BigSet001490|Contig2082	JA induced	401.7	724.3	407.3	600.0	gi|47104151|gb|BT012736.1|	dehydration-responsive methyltransferase
BigSet009445|Contig46793	JA induced	1439.3	2972.3	1489.7	2979.3	gi|134303382|gb|EF469866.1|	lipoxygenase
BigSet005254|Contig6830	JA induced	2054.7	1897.0	2742.7	2137.3	gi|76803809|gb|DQ176017.1|	fatty acid desaturase
BigSet009443|Contig10459	JA induced	4275.0	7289.7	6431.7	7478.3	gi|349726586|emb|FQ382796.1|	acetylornithine deacetylase
BigSet006265|Contig1259	JA induced	4404.3	6135.0	6952.7	6313.3	gi|14279757|gb|AF276072.1|AF276072	terpene biosynthesis
BigSet004137|Contig46799	JA induced	16160.3	12862.3	20781.0	13359.0	gi|134303541|gb|EF469930.1|	unknown
BigSet013655|Contig19209	JA induced	63.7	698.0	63.3	754.0	gi|17385637|dbj|AB035271.2|	WRKY tanscription factor
BigSet011625|Contig22613	JA induced	82.7	1010.7	77.7	1060.7	gi|17385637|dbj|AB035271.2|	WRKY tanscription factor
BigSet015246|Contig14217	JA induced	6055.7	17867.3	6000.7	18296.3	gi|313755449|gb|HM807397.1|	terpene biosynthesis
BigSet009822|Contig27628	JA induced	148.7	821.0	150.0	829.7	gi|225446436|ref|XM_002276515.1|	tyrosine aminotransferase-like
BigSet003179|Contig24022	JA induced	180.7	703.3	265.3	787.0	gi|225448450|ref|XM_002272959.1|	leucoanthocyanidin dioxygenase-like
BigSet003459|Contig13317	JA induced	906.7	2070.3	1118.7	1911.7	gi|224101152|ref|XM_002312126.1|	flavonol synthase
BigSet012756|c17430	JA induced	310.3	762.7	404.3	771.3	gi|225456646|ref|XM_002270776.1|	nitrate transporter
BigSet002647|Contig62013	JA induced	689.0	1884.7	644.7	1924.0	gi|353334517|gb|JN162091.1|	ethylene response
BigSet015044|HA89.CCFT6980.r	JA induced	349.7	765.3	373.0	817.0	gi|337730992|gb|JN021934.1|	UDP-glucose glucosyltransferase
BigSet009784|c3322	JA induced	183.7	194.7	232.3	204.0	gi|359493874|ref|XM_002284632.2|	NAC domain/JA responsive
BigSet005870|Contig50619	JA induced	2469.7	1338.0	2739.3	1428.0	gi|118486690|gb|EF147152.1|	dehydration-responsive/unknown
BigSet011107|Contig29284	JA induced	304.0	283.0	314.3	294.7	gi|359485053|ref|XM_002263963.2|	transcription factor bHLH35-like
BigSet008897|Contig27075	JA induced	57.7	158.3	59.0	157.3	gi|359480903|ref|XM_002267660.2|	4,5-DOPA dioxygenase extradiol-like
BigSet009361|Contig2244	JA induced	319.0	4138.7	405.7	4618.3	gi|168236|gb|M76546.1|HNNHPRG	extensin
BigSet015720|Contig24651	JA induced	1877.7	2847.7	1830.3	3029.3	gi|225447403|ref|XM_002281343.1|	terpene biosynthesis
BigSet010823|Contig1823	JA induced	366.0	1081.7	371.7	1088.0	gi|349718758|emb|FQ392474.1|	GDSL esterase/lipase
BigSet001802|Contig64002	JA induced	42.0	9545.7	42.0	10613.3	gi|359475595|ref|XM_002265014.2|	pathogenesis-related protein
BigSet008023|c13826	JA induced	7658.7	8800.0	7953.7	9107.7	gi|255544763|ref|XM_002513397.1|	lipoxygenase
BigSet010411|Contig51155	JA induced	443.3	1709.3	459.0	1868.3	gi|356550660|ref|XM_003543655.1|	WRKY tanscription factor
BigSet014029|Contig19943	JA induced	9155.7	10160.0	13340.3	10559.3	gi|22759722|dbj|AB091074.1|	protease inhibitor
BigSet005122|Contig53168	JA induced	1419.3	2481.7	1935.0	2813.0	gi|224140080|ref|XM_002323379.1|	unknown
BigSet007350|Contig51184	JA induced	2458.3	3633.7	3849.7	3637.0	gi|224056910|ref|XM_002299049.1|	protease inhibitor
BigSet014190|Contig52645	JA induced	2786.3	54217.7	2815.0	58074.3	gi|15149818|emb|AJ315591.1|	S-like Rnase
BigSet009994|Contig1737	JA induced	1400.7	1461.7	1646.7	1485.7	gi|160960513|emb|CU233019.1|	thiosulfate sulfurtransferase
BigSet000654|Contig45676	JA induced	135.7	239.3	137.0	250.7	gi|359495057|ref|XM_002267682.2|	flavonol synthase
BigSet000331|Contig63473	JA induced	2331.3	2363.3	2954.0	2467.7	gi|255566735|ref|XM_002524306.1|	tryptophan aminotransferase-related protein
BigSet008674|Contig29883	JA induced	129.0	422.0	140.0	445.0	gi|225425113|ref|XM_002273150.1|	phytosulfokine receptor
BigSet015539|Contig63491	JA induced	741.3	753.7	944.0	816.0	gi|356556367|ref|XM_003546450.1|	unknown
BigSet005816|ANN1312.gi_90463659	JA induced	705.3	1209.3	659.3	1262.7	gi|225319513|dbj|AK329892.1|	unknown
BigSet015320|RHA280.gi_22456554	JA induced	195.7	160.0	239.3	158.7	gi|147863303|emb|AM436910.2|	unknown
BigSet003407|Contig46260	JA induced	5477.0	9017.0	5957.3	9566.7	gi|147853964|emb|AM478339.2|	pathogenesis-related protein
BigSet009825|Contig23200	JA induced	233.3	58.7	252.7	56.0	gi|225317089|dbj|AK328854.1|	unknown
BigSet008539|Contig30697	JA induced	311.0	159.0	455.3	162.3	gi|145370791|dbj|AB195285.1|	serine carboxypeptitase
BigSet011813|Contig19537	JA induced	130.0	526.7	139.7	552.0	gi|145049631|gb|EF192422.1|	PnFL-2
BigSet013546|Contig7805	JA induced	302.0	153.3	323.3	154.7	gi|31581004|dbj|AP006373.1|	unknown
BigSet011042|Contig11312	JA induced	293.3	1415.3	397.0	1542.7	gi|147769272|emb|AM465119.2|	unknown
BigSet012333|Contig68580	JA induced	362.7	1236.0	413.7	1274.3	gi|356569044|ref|XM_003552669.1|	unknown
BigSet012226|Contig2204	JA induced	94.0	404.3	102.3	429.7	no hits	no hits
BigSet001894|Contig49678	JA induced	2010.7	3242.7	2015.3	3387.7	no hits	no hits
BigSet003571|Contig66896	JA induced	41225.7	56684.0	43452.7	61527.3	no hits	no hits
BigSet006908|Contig18452	JA repressed	-2423.7	-687.7	-1412.0	-3144.0	gi|225321388|dbj|AK319176.1|	GDSL esterase/lipase
BigSet013248|c3084	JA repressed	-2170.0	51.7	-7087.0	-5907.7	gi|7025492|gb|AF230332.1|	expansin/cell wall
BigSet005803|Contig2090	JA repressed	-976.0	143.7	-220.0	-344.7	gi|307746707|dbj|AB522639.1|	acyltransferase
BigSet000427|Contig18653	JA repressed	-443.0	42.3	-45.0	-102.7	gi|359476528|ref|XM_002268057.2|	GDSL esterase/lipase
BigSet010200|Contig63081	JA repressed	-1402.0	15.3	-523.7	-261.7	gi|224053990|ref|XM_002298038.1|	HOTHEAD-like/FAD containing oxidoreductase
BigSet010604|Contig3836	JA repressed	-1428.7	-455.3	-1271.3	-519.3	gi|225321049|dbj|AK325260.1|	GDSL esterase/lipase
BigSet005414|c18183	JA repressed	-305.7	54.7	-427.0	-618.0	gi|224063492|ref|XM_002301135.1|	poly-A binding
BigSet005305|HA89.CCFS1852.f	JA repressed	-798.3	-63.7	-347.0	-97.0	gi|359474261|ref|XM_003631378.1|	HOTHEAD-like/FAD containing oxidoreductase
BigSet010104|Contig38249	JA repressed	-763.7	-459.3	-166.3	-363.3	gi|47104289|gb|BT012874.1|	GDSL esterase/lipase
BigSet007769|Contig29203	JA repressed	-2464.7	-1519.3	-14460.0	-13185.0	gi|157058853|gb|EU116342.1|	germin-like/auxin-binding
BigSet010232|Contig3574	JA repressed	-6978.3	-1686.0	-4569.7	-6463.3	gi|48375045|gb|AY554167.1|	unknown
BigSet007894|Contig5130	JA repressed	-125.7	-146.7	-133.7	-1383.3	gi|3402281|emb|AJ000997.1|	unknown
BigSet002740|Contig339	JA repressed	-3645.0	-317.0	-3046.3	-4518.7	gi|224119789|ref|XM_002318127.1|	acyl carrier
BigSet003755|Contig46171	JA repressed	-897.3	21.3	-398.7	-412.0	gi|225314248|dbj|AK320994.1|	unknown
BigSet000334|Contig1708	JA repressed	-2302.7	-2155.7	-1936.0	-537.0	gi|356546608|ref|XM_003541669.1|	GDSL esterase/lipase
BigSet012058|Contig66017	JA repressed	-2161.3	-3.3	-575.7	-336.0	gi|225318181|dbj|AK323338.1|	unknown
BigSet000648|RHA801.gi_22310605	JA repressed	-3352.7	-820.3	-2386.7	-3368.0	gi|1794144|dbj|AB000505.1|	unknown
BigSet007515|Contig22640	JA repressed	-104.3	-15.7	-91.7	-1418.0	gi|51997114|emb|AJ783397.1|	unknown
